# Cerebrovascular Diseases in Workers at Mayak PA: The Difference in Radiation Risk between Incidence and Mortality

**DOI:** 10.1371/journal.pone.0125904

**Published:** 2015-05-01

**Authors:** Cristoforo Simonetto, Helmut Schöllnberger, Tamara V. Azizova, Evgenia S. Grigoryeva, Maria V. Pikulina, Markus Eidemüller

**Affiliations:** 1 Helmholtz Zentrum München, Department of Radiation Sciences, Neuherberg, Germany; 2 Southern Urals Biophysics Institute (SUBI), Ozyorsk, Chelyabinsk Region, Russia; University of Hawaii Cancer Center, UNITED STATES

## Abstract

A detailed analysis of cerebrovascular diseases (CeVD) for the cohort of workers at Mayak Production Association (PA) is presented. This cohort is especially suitable for the analysis of radiation induced circulatory diseases, due to the detailed medical surveillance and information on several risk factors. The risk after external, typically protracted, gamma exposure is analysed, accounting for potential additional internal alpha exposure. Three different endpoints have been investigated: incidence and mortality from all cerebrovascular diseases and incidence of stroke. Particular emphasis was given to the form of the dose-response relationship and the time dependence of the radiation induced risk. Young attained age was observed to be an important, aggravating modifier of radiation risk for incidence of CeVD and stroke. For incidence of CeVD, our analysis supports a dose response sub-linear for low doses. Finally, the excess relative risk per dose was confirmed to be significantly higher for incidence of CeVD compared to CeVD mortality and incidence of stroke. Arguments are presented for this difference to be based on a true biological effect.

## Introduction

Circulatory diseases are the leading cause of death worldwide [1]. The most common circulatory diseases are ischemic heart diseases (7.3 million deaths in 2008) and stroke (6.2 million deaths in 2008), each about as frequent as cancer (7.6 million deaths in 2008) [2]. These high absolute numbers reveal the impact of any relative risk enhancement due to radiation.

After radiotherapy, circulatory complications [3, 4], including cerebrovascular diseases [5, 6] are known as late effects. Traditionally, this is interpreted as a deterministic effect [7]. However, epidemiological studies, initially of the atomic bomb survivors [8], have also revealed risk at lower doses [9]. Several effects of low doses on the circulatory system have also been found by animal experiments [10–12]. The precise risk for circulatory diseases from low dose radiation as well as the main biological mechanisms remain unknown. However, atherosclerosis seems to play a major role [13] to which premature senescence may contribute, as indicated by cell experiments [14].

The Mayak Workers Cohort provides one of the most important sources to assess the risk of radiation. The present analysis is based on the workers hired before 1973 at the main plants of the Mayak complex, which was founded in 1948 for the production of weapon grade plutonium. Full clinical records including regular medical examinations offer the possibility to study not only mortality but also incidence of various diseases. For the case of circulatory diseases, information on comparatively many non-radiation risk factors is of particular importance. The data are continuously extended and updated. Results are published regularly, analyzing the dose response with categorical and linear models [15–20]. However, an analysis of the dose response as minute as possible is advisable, in view of the high frequency of circulatory diseases and the absence of an understanding of the mechanisms. A close analysis of the dose response and its age dependence has already been performed for mortality of circulatory diseases in the atomic bomb survivors [21] and for incidence and mortality of ischemic heart diseases, the most frequent circulatory diseases, in the Mayak Workers [22]. This study supplements previous work by a comprehensive analysis of the second most frequent circulatory diseases, cerebrovascular diseases, after external gamma exposure in the Mayak Workers. As it is impossible to pin down the true model of the dose response without precise knowledge of the biological mechanisms, we compiled results from fits of several models with multi-model inference [23, 24]. With this method, the result does not rely on a single model alone. This leads to more reliable confidence intervals, in particular in regions with substantial differences between models as commonly observed at the boundaries of the data set, for example at low doses.

In addition, our analysis differs from [20], in that we restricted the analysis to a subcohort with negligible internal exposure for incidence of CeVD and modeled the baseline analytically. Moreover, we analyzed stroke incidence, which has been done in ref. [16] for the last time but on a considerably smaller cohort. Stroke is the most frequent fatal form of CeVD and its analysis may help to understand the observed difference in radiation risk between incidence and mortality of CeVD.

## Materials and Methods

### Materials: Cohort definition

This section focuses on the characteristics of the cohort most important for the analysis. Complementary information can be found in refs. [17, 20], a more detailed description on data collection and cohort definition is given in refs. [25, 26].

#### Study cohort

The study cohort is based on the nuclear workers at Mayak PA, close to the town of Ozyorsk. All workers of the main facilities (reactors, radiochemical and plutonium plants) employed before 1973 were included from their first day of occupation. Because of acute radiation syndrome 43 persons were excluded, as well as 57 persons because of peculiar variations in the measured internal exposure, which may be indicative for unusual uptake scenarios.

The cohort includes 18,797 workers of which 4,741 are women. However, for reasons outlined below, different subgroups were analyzed in this work. The number of persons, person years and cases for which the different analyses were performed, are summarized in Table 1.

**Table 1:** Number of persons, person years and cases for the different analyses performed in this work.

	CeVD incidence (reactor)	CeVD incidence (main plants)	Stroke incidence	CeVD mortality
Persons	2,166	9,728	18,797	18,797
Person years	34,331	148,868	440,832	732,065
Cases	1567	7,174	1613	1551

For CeVD incidence, the cohort was restricted with respect to certain ages and calendar years and the main analysis refers only to reactor workers.

Mean age of first employment is 25 (5% and 95% percentiles: 17; 45) years. First CeVD incidence occurred at a mean age of 57 (44; 72) years, first stroke incidence at a mean age of 66 (43; 86) years and the average age of CeVD mortality is 71 (50; 88) years.

#### Endpoints

Diagnostic methods for CeVD have improved over time. For this study, however, all cases have been verified according to clinical symptoms and signs. Cerebrovascular diseases are specified by the ICD-9 codes 430–438 for incidence and mortality (International statistical Classification of Diseases and related health problems). Stroke is defined by the ICD-9 codes 430–432, 434 and 436. While 71% of first stroke cases refer to ICD-9 code 434, occlusion of cerebral arteries, first CeVD incidence was diagnosed already before in almost all cases and ICD-9 code 437.0, cerebral atherosclerosis, was assigned in 96% of the cases. Thus, first CeVD incidence refers to a chronic disease in almost all cases and by that differs substantially from the acute disease of stroke.

For most of the deaths in Ozyorsk, full clinical documentation is available. In order to focus on atherosclerotic induced cerebrovascular diseases, deaths from Ozyorsk residents were considered only if the underlying disease was coded as 437.0. This specification excluded less than 2% of all CeVD mortality cases.

#### Radiation exposure and dosimetry

Based on film badge readings, each worker has been assigned annual dose estimates from external gamma radiation. We apply the Mayak Worker Dosimetry System 2008 [27]. A short summary of the dosimetry system with an emphasis on the latest improvements and their impact on the analysis of lung cancer can be found in ref. [28]. The mean total dose from external gamma radiation is 0.59 (5% and 95% percentiles: 0.0; 2.7) Gy. As medical assistance and safety measures improved over time, major exposure typically occurred during the first years of employment. Accordingly, the average of the median ages at exposure is 29 (19; 49) years and the mean duration of half exposure is 5 (< 1; 19) years.

In addition to external exposure, many workers have incorporated Plutonium. For about one third of the workers, Plutonium-239 body burden could be evaluated from results of biophysical examinations and autopsy data [27, 29]. The mean cumulative absorbed liver dose from internal alpha radiation adds up to 0.35 (5% and 95% percentiles: 0.006; 2.4) Gy. As measurements were often performed in relation to the potential intake, the average of the internal dose in the full cohort can be expected to be markedly lower. So far, dose estimates to the blood vessels are not provided. In spite of the measurements, estimates for internal exposure are subject to rather high uncertainties [30], and the analysis is aggravated by the assignment of measurements below the limit of detection [20] and the fact that first measurements have often been performed years or decades after first employment. Therefore, we will not pursue the analysis on internal doses already performed in ref. [20].

To partially correct for the effect of internal doses when analyzing external gamma doses, surrogate classes of internal exposure have been developed by the Southern Urals Biophysics Institute (SUBI) Laboratory of Epidemiology: Based on work place and year of employment, each worker is classified into one of six categories corresponding to the presumed magnitude of internal dose [31, 32]. Thus, the correction can be applied to the full cohort and the surrogate classes are free from the selection bias related to the measurements.

#### Follow-up

For stroke incidence and CeVD mortality, the begin of follow-up for each person is given by the date of first employment. The end of follow-up is defined by the earliest of: December 31, 2008, date of first stroke/death or date of last information. The latter refers to the availability of clinical data in Ozyorsk. An exception is mortality among migrants for whom information after migration from Ozyorsk was provided by the SUBI Laboratory of Epidemiology up to and including 2005.

**Fig 1:**
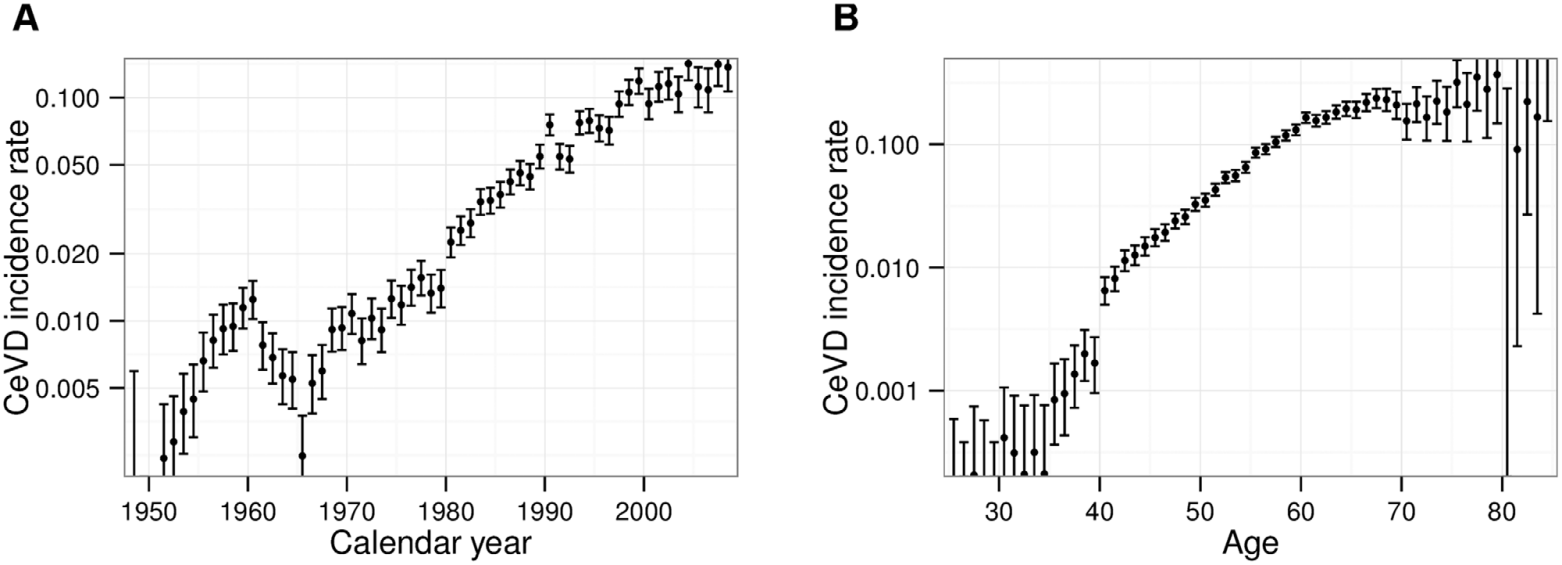
Crude CeVD incidence rates and 95% confidence intervals of the full cohort. (A) Crude rates for different calendar years. (B) Crude rates for different ages. The crude rate is the number of cases per person years.

For incidence of CeVD, an analogous definition of follow-up was applied but with several exclusions: Internal dose is known to be a significant risk factor for CeVD incidence [17, 20]. Separation of risks induced by internal and external exposure turned out to be difficult, in particular when analyzing in detail the dose-response relationship or the time dependence of the risk. One reason may be the existence of some correlation between external and internal dose together with the uncertainties associated with internal doses. (The Spearman’s rank correlation coefficient evaluates to 0.5 for those workers with measurement of internal dose.) Moreover, the assumption that the dual stressors of external and internal dose act multiplicatively on the radiation risk, may fail. These problems were avoided by restricting the main analysis to reactor workers for whom internal dose is expected to be negligible. In doing so, the incidence analysis becomes free of confounding due to internal exposure. On the other hand, exclusions of workers at radiochemical or plutonium plants are not necessary for stroke incidence nor CeVD mortality as the effect of internal exposure turned out to be insignificant for these endpoints. In addition, person years were restricted to the time after end of the year 1960. In the left panel of Fig 1 the number of incident cases per person years is plotted for different calendar years. In opposition to the overall increase due to aging of the cohort there is a peak around 1960. The underlying reasons for this peak are unknown to the authors but a similar behavior has already been observed in incidence of ischemic heart diseases [22] and chronic bronchitis [33]. In both publications, person years before 1960 have been excluded. Remaining features in the calendar year dependence were modeled with a linear spline, see section A.2.1 in S1 Appendix. Finally, all person years below an age of 40 years were excluded. In this regime, the observed number of cases is markedly lower than the already low number that can be expected from extrapolation from higher ages, see the right panel of Fig 1. Exclusion of these relatively young ages has little impact on the number of cases in the cohort. We demanded the age dependence to be differentiable, see section A.2.1 in S1 Appendix. Despite these exclusions, the number of cases is similar for the different endpoints due to the high rate of CeVD incidences, see Table 1.

#### Ethics Statement

This record-based epidemiological study did not require any contact with the cohort members. Information was anonymized and de-identified prior to analysis. The project was reviewed and approved by the Institutional Review Board of the Southern Urals Biophysics Institute (SUBI).

### Methods: Statistical analysis

Data were analyzed using an individual likelihood analysis with the models for baseline and radiation effect described below. The total likelihood *L* is obtained by a product of the individual likelihoods. The minimum deviance (−2 ln *L*) and the corresponding parameter values were determined using MINUIT [34]. Tests of statistical significance (with significance level *P* = 0.05) and 95% confidence intervals were calculated from likelihood ratios assuming a *χ* ^2^-distribution for the deviance.

#### Baseline data

One of the strengths of the Mayak Workers Cohort is the comparatively large number of covariables that can be accounted for. First, there is information on the dates of birth, employment and emigration from Ozyorsk. The latter is important due to the better medical surveillance in the closed town of Ozyorsk and a potentially different lifestyle. Moreover, while for most Ozyorsk residents full clinical data are available, information on emigrants is limited to death certificates. Secondly, categorical data on smoking (non-smoker, smoker and ex-smoker, unknown) and drinking status (non-drinker, drinker and ex-drinker, unknown), on body mass index (< 18.5 kg/m^2^, normal, ≥ 25 kg/m^2^, unknown), blood pressure (normal, above 140/90 mmHg, unknown), graduation (primary education, secondary education, secondary special education, entered higher education, unknown) and on work plant (work only in reactor, only in radiochemical plant or reactor, at least for some time in plutonium plant) are available. While for body mass index and blood pressure, information from the pre-employment medical examination was used, data on smoking and drinking behavior were gathered by interviews at several medical examinations. Non-smokers/non-drinkers are workers who always have claimed that they had never been a smoker/drinker.

The analytic baseline function in section A.2.1 in S1 Appendix may account for all of these covariables. However, only parameters significantly deviating from zero were included. While the baseline function was fitted separately for each gender, a joint dose response for males and females was applied in order to gain statistical power.

#### Time dependence of the excess risk

Different time scales might influence progression of the disease. To disentangle the relevant ones, we independently tested modification of the dose response with the time variables age attained (*a*), age at median exposure (*e*), time since median exposure (*tse*) and duration of half exposure (*dhe*). Age at median exposure is the age at which half of the hitherto accumulated dose was received, time since median exposure is *tse* = *a* − *e*. In order to suppress years with minor exposures, *dhe* was applied instead of the duration of the whole exposure. It is defined to be the time span from end of the first to beginning of the last quartile of the hitherto accumulated dose. An illustration can be found in ref. [22].

For each time variable we tested modification of the excess relative risk from external exposure (*ERR*_ext_, defined in eq. (A4) in S1 Appendix) with two different functions. The first is exponential in time:

$$ERR_{\text{ext}}\left(d,v\prime \right)=ERR_{\text{ext}}\left(d\right)\;\exp\left(\mu \prime v\prime \right)$$ (1)

where *d* refers to dose, *v*′ may be any of ln(*a*/60), *e* − 30, *tse* − 30 and *dhe* − 5 and *μ*′ is a free parameter. All time variables are given in units of years. Secondly, we tested with the logistic function, a (smoothed) step function:

$$ERR_{\text{ext}}\left(d,v\right)=ERR_{\text{ext}}\left(d\right)\frac{1}{2}(1\pm \tanh(v-\mu ))$$ (2)

with *v* any of *a*, *e*, *tse* and *dhe*. The step function jumps from zero to one (resp. from one to zero) in the region around *μ*. We tested for both signs in Eq (2), i.e. the risk may vanish either for small or for large values of *v*. In the analysis of the time dependence, the Linear-No-Threshold (LNT) dose-response relationship is assumed (defined in eq. (A6) in S1 Appendix).

#### Modification by other risk factors

To analyze possible interactions between radiation and other risk factors we modified the LNT dose response *ERR*_ext_ by a factor $\exp(\psi_{\text{cat}}^{\text{mod}})$. Here, the value of $\psi_{\text{cat}}^{\text{mod}}$ depends on gender, smoking or drinking behaviour, body mass index or blood pressure. To ensure separation of baseline and radiation risk modification of a risk factor, the corresponding variable in the baseline template was also released even if it had previously been shown not to be a significant covariable.

#### The dose-response relationship

For the dose-response relationship, we applied up to ten different functions, which can be found in eq. (A6) and are sketched in Fig. A1 in S1 Appendix. To select the best models, we performed a series of likelihood-ratio tests between nested models (see Fig. A2 in the appendix of [22]). Starting from the models with fewest parameters, comparison was always performed to the currently best model in a series of nested models. Non-nested models were compared according to the Akaike Information Criterion (*AIC*), defined by the sum of the deviance plus twice the number of parameters [35, 36].

## Results

### Analysis with linear dose response and without effect modification

Results of the analyses within the LNT framework are presented in Table 2. We tested for several lag times as it is unknown how long it takes from a low dose rate exposure to an effect on CeVD risk. In addition to the main CeVD incidence analysis focusing only on reactor workers, workers from all main plants were included in a second analysis. Independent of this choice and for all lag times, a highly significant risk for CeVD incidence was observed. Within the reactor workers, the best fit, i.e. the lowest deviance, was obtained for a vanishing lag time (Δ*dev* ≡ 0, *P* value for radiation effect *P* < 0.001) and for 10 years (Δ*dev* = −0.2, *P* < 0.001) while lag times of 20 and 30 years were disregarded based on their deviance (Δ*dev* = 9.5 and Δ*dev* = 19.8, respectively). In the second analysis, i.e. analyzing workers from all main plants, the lag time was applied to the surrogate categories of internal exposure in addition to the gamma dose. Hence, the goodness of fit cannot be used to infer the duration from a gamma exposure to an effect in CeVD incidence. In contrast to CeVD incidence, no association of external doses with risk could be established for stroke incidence (*P* > 0.3) and CeVD mortality (*P* > 0.4). While the excess relative risks per dose (*ERR*_pd_) for stroke incidence and CeVD mortality are compatible, the observed *ERR*_pd_ is much higher for CeVD incidence.

**Table 2:** Excess relative risk per dose [Gy^−1^] and 95% confidence intervals for external gamma radiation.

lag time [y]	0	10	20	30
CeVD inc. (reactor)	0.33 (0.19; 0.50)	0.35 (0.20; 0.53)	0.33 (0.16; 0.52)	0.32 (0.11; 0.58)
CeVD inc. (main plants)	0.37 (0.30; 0.45)	0.39 (0.31; 0.46)	0.33 (0.25; 0.41)	0.28 (0.19; 0.38)
Stroke incidence	0.01 (-0.06; 0.08)	0.00 (-0.06; 0.07)	-0.01 (-0.07; 0.06)	-0.03 (-0.10; 0.04)
CeVD mortality	0.02 (-0.05; 0.10)	0.03 (-0.04; 0.10)	0.04 (-0.03; 0.12)	0.02 (-0.05; 0.11)

The LNT model was applied using various lag times.

### Time dependence of the excess risk

Next, we compared impact of the time variables age, age at median exposure, time since median exposure and duration of half exposure. For each time variable, we tested modification of the excess relative risk (ERR) both with an exponential and with a step function according to Eqs (1) and (2), assuming no lag time.

For CeVD incidence, we observed that the risk is enhanced for relatively young ages: Modifying the excess relative risk exponentially with attained age, the deviance was improved by 4.8. The *ERR*_pd_ of 0.25 (95% confidence interval: 0.10; 0.43) Gy^−1^ at an age of 60 years declines strongly with attained age, *μ*′ = -3.7 (-7.7; -0.3), see Eq (1). An even better improvement of the deviance, by 6.1 points (*P* = 0.01), was obtained when modifying the ERR by a decreasing step in age attained instead of an exponential decline. The best fit was obtained for an *ERR*_pd_ of 0.39 (0.24; 0.59) Gy^−1^ that drops down to zero around an age of *μ* = 64.4 (63.2; 70.1) years, according to Eq (2). Deviances and parameters can also be found in S1 and S3 Tables. Modification with time variables other than attained age did not yield a significant improvement of the fit.

Interestingly, for stroke incidence we observed a significant risk for relatively young ages, too. Although the analysis without effect modification did not show any improvement in deviance compared to the baseline, the dose effect became significant when modifying with a step in attained age (*P* value for radiation effect modified by age attained, *P* = 0.03). For a decline at attained age of 64.2 (57.8; 69.5) and an *ERR*_pd_ of 0.14 (0.03; 0.28) Gy^−1^ the deviance was improved by 7.4 compared to the baseline, see also S2 and S3 Tables. In general, significant modifications of *per se* insignificant effects may result from multiple testing. In view of the CeVD incidence result, however, this finding appears plausible. It was the only significant modification for stroke incidence and we did not observe any for CeVD mortality.

In addition to excess relative risk models, an excess absolute risk model (see eq. (A5) in S1 Appendix) was also tested allowing for modification by age attained according to Eqs (1), (2). For none of the endpoints, however, there was any significant dose response with improved deviance compared to the ERR model with the same modification.

### Modification by other risk factors

No significant modification could be observed in CeVD incidence (see S4 Table). Hence, this analysis was not performed for stroke incidence nor CeVD mortality.

### The dose-response relationship

**Fig 2:**
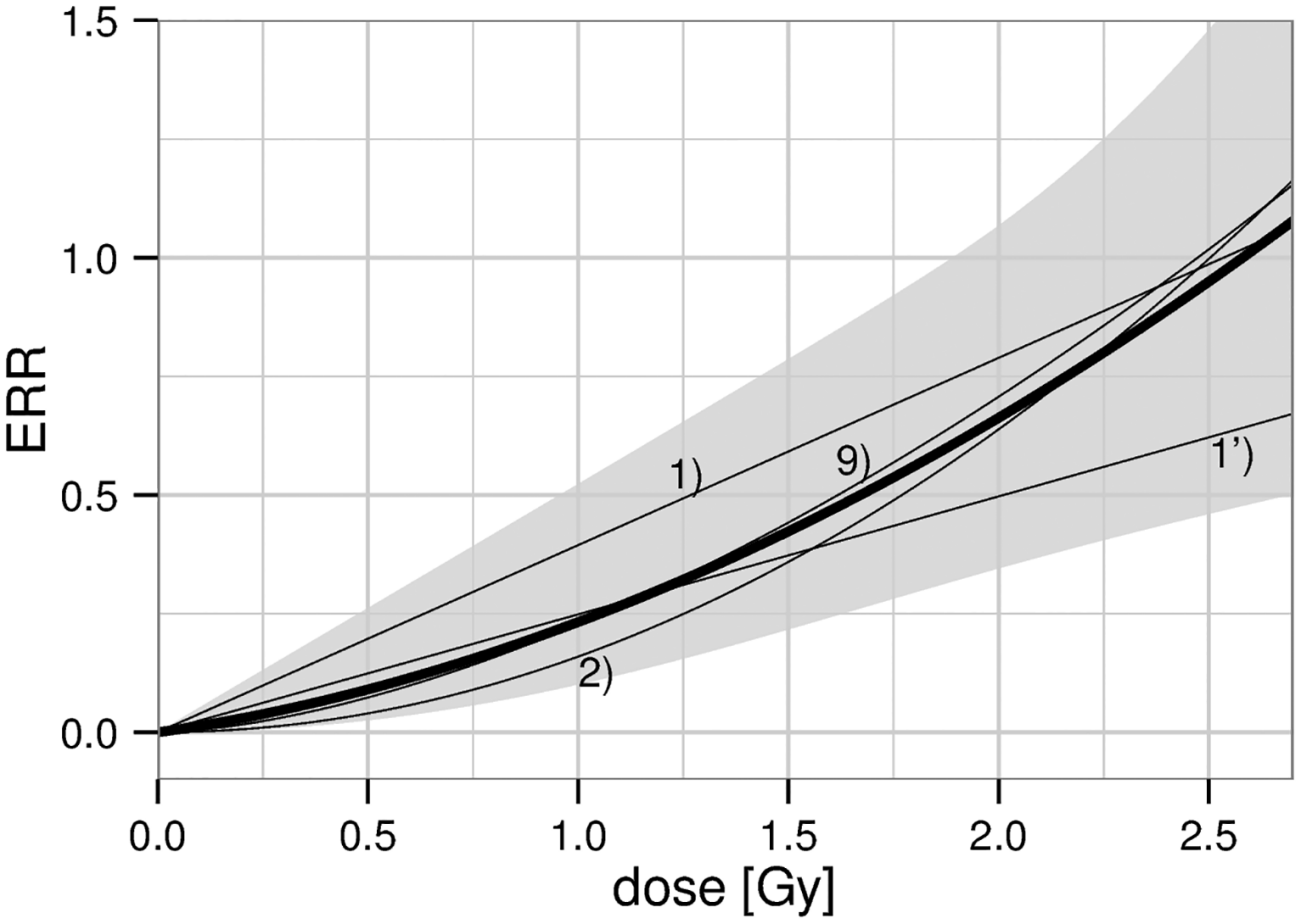
Excess relative risk for CeVD incidence at an attained age of 60 years. Thin lines correspond to the final, non nested models, numbered according to Fig. A1 in S1 Appendix: 1) LNT model with step in age attained, Akaike weight 23%; 1’) LNT model exponential in age attained, Akaike weight 12%; 2) Quadratic model with step in age attained, Akaike weight 53%; 9) Sigmoid model with step in age attained, Akaike weight 11%. The thick line with 95% confidence band represents the result of multi-model inference.

For the CeVD incidence analysis, motivated by the results above, different functions of the dose-response relationship have been applied with modification for age attained in addition to the unmodified analysis. In any case, the quadratic model was observed to describe the data somewhat better compared to the linear model. In addition, the sigmoid function passed the likelihood-ratio test. However, as the inflection point is far beyond the doses relevant for the Mayak Workers Cohort, the sigmoid function converges to a power law with power *λ*_1_ = 1.6 (1.0; 2.5), thus being similar to the linear-quadratic model. Deviances of all dose-response models can be found in S1 Table; parameter values are presented in S3 Table only for the models that passed the likelihood-ratio test. For CeVD incidence, these models are presented in Fig 2. Related Akaike weights, which are proportional to exp(−0.5*AIC*), are provided in the figure legend. The result of multi-model inference is shown with a thick line. The 95% confidence interval was obtained from a weighted superposition of the likelihood distributions of the models contributing to the multi-model average. In doing so, each likelihood distribution was approximated by a split-normal distribution of the observed *e* ^−2^ likelihood interval. Results of multi-model inference for preselected values of age attained and dose can be found in Table 3. Due to the strong impact of sub-linear models, in particular the quadratic model, values for the excess relative risk are below the estimate of 0.33 (0.19; 0.50) Gy^−1^ from the fit with the linear, unmodified dose response, Table 2.

**Table 3:** Values of the excess relative risk and 95% confidence intervals for CeVD incidence calculated with multi-model inference.

Age attained	50 years	60 years	70 years
0.2 Gy	0.04 (0.00; 0.13)	0.03 (0.00; 0.10)	0.00 (0.00; 0.05)
0.5 Gy	0.11 (0.03; 0.32)	0.09 (0.03; 0.26)	0.01 (0.00; 0.13)
1.0 Gy	0.26 (0.10; 0.63)	0.23 (0.10; 0.52)	0.02 (0.01; 0.28)

For incidence of stroke and CeVD mortality the linear dose response is less pronounced and insignificant, respectively. Deviations from linearity are hence more difficult to detect. Thus, we restricted ourselves to models of the dose-response relationship with at most two parameters. For stroke incidence, none yielded a significant dose response without effect modification. Repeating the stroke analysis of the dose response but modifying with a decreasing step in age attained, most models significantly improved the deviance compared to the baseline, see S2 Table. The linear, the quadratic and the smoothed step model were selected by the likelihood-ratio test. For all models, however, we found two minima in the deviance corresponding to two different sets of parameter estimates. In the first minimum, the step in attained age occurs right before an age of 50 years while it occurs at an age of approximately 60 years in the second minimum, see S3 Table. For the linear model, the best fit was obtained for a step in age attained above 60 years, while a decrease before age of 50 years was preferred for the quadratic model. In case of the smoothed step model, both sets of parameter estimates yielded essentially the same deviance. The smoothed step model with the step at about 2 Gy, modified with a decreasing step in age attained at 49 or 59 years was favored by the *AIC*. However, independent of the choice of the minimum, this model is based on a few workers at the boundary of the data set and should be interpreted with caution. There are only 50 cases with doses above 2 Gy and an age below 59 years. Additionally, it should be noted that this step in dose yielded a very poor fit without modification in attained age. In the analysis on CeVD mortality no dose-response model reached significance.

## Discussion

### Strengths and limitations

The Mayak Workers Cohort constitutes a large cohort with already 60 years of follow-up. At least for the time of residence in Ozyorsk, follow-up is almost complete, medical surveillance is assured by regular examinations and disease assignment is facilitated by the existence of full clinical records. Quality control checks have been conducted to ensure the accuracy of the data [26]. The only information used from the time after the workers migrated away from Ozyorsk is date and cause of death for the mortality analysis. Dose estimates for external gamma exposure are based on continuous individual measurements. Additionally, a large part of the cohort has been exposed to incorporated Plutonium. To avoid bias, we excluded this part when internal doses contribute significantly to the risk (see the Materials section). Doses from other sources, including neutrons or medical X-rays are substantially lower and unlikely to affect our results.

Many risk factors affect cardiovascular risk and may bias radiation risk estimates if not properly taken into account. In the Mayak Workers Cohort, information on many risk factors have been collected. Still there could be residual confounding, either due to a too rough categorization or due to missing information on several risk factors. In particular, labor conditions could be related to cardiovascular health, for example because of stress, lack of exercise or shift work [37–40]. Information on these risk factors was not available. However, we correct for date of first employment, for plant and graduation, each possibly being a surrogate for labor conditions.

### Comparison to previous study

Compared to the recent study, which has been extended to workers hired before 1983 [20], a quite different methodology was adopted here. Instead of a stratified baseline, an analytical baseline function was constructed, continuous in age and calendar year. Baseline functions were fitted separately for each gender as many parameters turned out to be different. To avoid bias, all significant covariables were accounted for at the same time. On the other hand, we were as parsimonious as possible with the number of baseline parameters in order not to unnecessarily inflate the error bars. For the main CeVD incidence analysis, a subcohort was defined, devoid of certain potential biases (see the Materials section) but still large enough so that conclusions on the dose-response relationship for external radiation exposure and its effect modifications could be derived. In particular, the subcohort was restricted to reactor workers of whom the majority had never occupationally been exposed to alpha particle emitters, and doses were low for the others so that they could be neglected. Finally, graduation was added as an additional covariable. Potentially, it could correct not only for some aspects of lifestyle but also for individual working conditions.

For CeVD incidence, these differences in the analysis lead to somewhat different conclusions although the estimates of the excess relative risk per dose are highly consistent. When fitting a linear dose-response function and including workers from all main plants, we observed *ERR*_pd_ = 0.37 (0.30; 0.45) Gy^−1^, see Table 2. This fits nicely to *ERR*_pd_ = 0.34 (0.26; 0.44) Gy^−1^, the result of [20] when taking internal dose into account for those persons with measurement. It is interesting to note that these results are only marginally consistent to *ERR*_pd_ = 0.46 (0.37; 0.57) Gy^−1^, corresponding to the result of [20] without correction for internal exposure. We could reproduce the higher *ERR*_pd_ when we skipped the correction for internal doses (eq. (A4) in S1 Appendix) but, instead, fully took into account the baseline risk dependence on work plant. (When correcting for internal doses, the baseline risk for workers at the Plutonium production plant was not significantly different to the baseline risk for workers at the reactors. Hence, no covariable was included for work at the Plutonium production plant in this case.) These observations emphasize the importance of a correction for internal exposure. As explained in the Methods section, in order to be free from potential bias due to internal exposure, our main analysis refers only to reactor workers. As a result, the excess risk in our main analysis is even lower, *ERR*_pd_ = 0.33 (0.19; 0.50) Gy^−1^, see Table 2. This value is smaller compared to the result for reactor workers found in table 2 of ref. [20] but well within the error bars. In addition, a significant decrease in risk was found with increasing age attained. This modification was already visible as a marked log-linear trend in ref. [20]. However, their use of categories in age attenuates the gradient of this decline compared to our best estimate. The exponential decline is reinforced in our study by the exclusion of young ages. Finally, the reservations expressed in [20] concerning competing causes of death are of no concern to our analysis, which is based on individual likelihood and an analytical baseline model. The most important, qualitative difference to previous studies, however, relates to the shape of the dose-response relationship. While the categorical analysis of ref. [20], Fig. 3, strongly points to a linear dependence, our analysis prefers a quadratic dose-response function, see Fig 2 and S1 Table. This difference emerges as a benefit from focusing exclusively on reactor workers. We could reproduce the LNT as the best model when including workers from all main plants, correcting for work plant but not for internal doses. However, the quadratic dose response was favored when including workers from all main plants and correcting for internal doses using surrogate categories. Therefore, the linearity observed in ref. [20] might be a consequence of insufficient correction for risk of internal doses. In this case, this linearity might result as a combination of a sub-linear response to external gamma doses, as observed in the present study, together with a supra-linear response to internal alpha dose, as observed in ref. [20].

Stroke has not been analyzed in ref. [17, 20] but has been dealt with in ref. [16], with no evidence for an effect in the linear, unmodified analysis. Due to updates and extensions of the cohort, the number of cases has more than doubled since then. Nevertheless, their result agrees with our findings. There is also agreement in the non-significance of any radiation effect in CeVD mortality [20].

### Comparison to other cohorts

The literature on epidemiological studies of cerebrovascular diseases after low doses and low dose rate exposures to ionizing radiation has been reviewed in [9]. After that publication, CeVD mortality has been analyzed in refs. [41, 42]. There are, however, only few epidemiological studies on incidence. The only publication known to the authors providing an estimate on the excess relative risk per dose for CeVD incidence (as a whole), is based on Chernobyl emergency workers [43]. For the analysis with linear dose response and without effect modification, they find *ERR*_pd_ = 0.45 (0.11; 0.80) Gy^−1^, i.e. our result, Table 2, is well included in their error bars. Their modification by duration of exposure could, however, not be confirmed in the present study. Instead, we observed the risk to be higher below an age of about 60 years. In addition, several models of the dose response have been tested and a quadratic dose-response relationship was found to be preferable, see S1 Table.

Incidence of stroke was analyzed for the atomic bomb survivors in [44], wherein “stroke II” corresponds to the ICD-9 codes used for stroke within the present study. Their result of *ERR*_pd_ = 0.07 (-0.08; 0.24), taking into account smoking and drinking, is in agreement to our null result, Table 2. For stroke, neither results for the dose-response relationship nor effect modification are presented in ref. [44]. We found significant risk only for persons below an age of about 60 years. In a more recent study on stroke in atomic bomb survivors [45] radiation was significantly associated only to hemorrhagic strokes, which typically develop a few years earlier compared to the more common ischemic strokes.

Our results on CeVD mortality, Table 2, are consistent with 95% confidence with all studies listed in table 2 of the review [9] investigating CeVD as the underlying cause of death as well as with [41, 42]. In particular, they are marginally consistent with the significant excess relative risk per dose of 0.09 (0.01; 0.17) Gy^−1^ observed in the atomic bomb survivors [46] and well within the 90% error bars of the relatively large *ERR*_pd_ = 0.43 (-0.10; 1.12) observed in nuclear workers in UK [47]. Moreover, results from the atomic bomb survivors on CeVD mortality support a quadratic dose-response relationship and a higher risk for persons below an age of 60 years [46]. These findings fit nicely to our results on incidence but for mortality we could not address these issues due to the lack of significance of any effect.

Despite the overall very good consistence within the literature, the results of the Mayak Workers Cohort were found to introduce heterogeneity into a meta-analysis [48]. This finding, however, was based on a comparison of CeVD incidence to mortality.

### ERR in incidence vs. mortality

It is often assumed that for a certain disease the excess relative risk is identical for incidence and mortality. Typically, the probability to die from a certain disease is independent of whether the disease was induced by radiation. In this case, a relative increase in the number of incidences (at a given age) leads to the same relative increase in mortality [49]. Thus, the above assumption can be justified (at least for the case of an age independent excess relative risk). For the Mayak Workers Cohort, however, this equality is violated as the ERR is higher for CeVD incidence compared to CeVD mortality [16, 17, 20].

In ref. [48] this difference was hypothesized to arise from problems with loss of follow-up in the mortality cohort and from lower diagnostic accuracy associated with death certificate reporting. This reasoning now has to be abandoned: Follow-up for stroke incidence as well as for CeVD incidence are both restricted to the time of residence in Ozyorsk, for which full clinical data are available. Moreover, for the majority of first stroke cases, correct diagnosis is essential for the choice of the treatment: only for 15% of first strokes registered in the Mayak Workers cohort, the date of diagnosis corresponds to the date of CeVD death. Nevertheless, the results for stroke incidence are inconsistent with CeVD incidence but consistent with CeVD mortality, see Table 2.

In the analysis of ischemic heart diseases, we had argued that the longer latency for mortality compared to incidence might not allow for detection of the excess in the mortality risk [22]. This rationale, however, cannot be applied here. The excess relative risk is enhanced especially for relatively young ages—in incidence already a lag time of 20 years was disregarded by the fit, see the first paragraph of the Results section. The median of the time from first CeVD incidence to death from CeVD amounted to 16 years. Taking this together, risk should be enhanced in mortality with a latency of at most 30 years after radiation exposure. Yet the excess relative risk turned out to be very small even for a lag time of 30 years in the mortality and stroke analyses, Table 2.

Thus we were led to the conclusion that the difference of the excess risk in CeVD incidence to stroke incidence and CeVD mortality may reflect the specific pathogenesis of radiation induced cerebrovascular diseases. Given the fact that the term “cerebrovascular diseases” covers several distinct disease entities, it might not be surprising if only some of them can be induced by radiation. For example, in the atomic bomb survivors hemorrhagic stroke was observed to be more frequent after exposure but not ischemic stroke [45]. Incidence is mainly related to chronic forms of CeVD while mortality refers mostly to acute forms of CeVD such as stroke [17]. Thus, to explain the difference of the excess risk in CeVD incidence to stroke incidence and CeVD mortality, it might be sufficient if radiation rather leads to chronic than acute forms of CeVD. In fact, for patients of radiotherapy, differences between radiation induced and spontaneous lesions have been recognized concerning size, sites and sonographic features [50]. Interestingly, radiotherapy induced plaques were found to be more fibrous and less inflammatory in a clinical trial [51]. Such plaques could be expected to be more stable than spontaneous plaques. In this case, radiotherapy induced plaques may result in acute forms of CeVD in comparatively few cases.

Finally, it should be noted that the difference of mortality to morbidity was already recognized as a major source of heterogeneity in ERR in a meta-study [52]. However, it is also important to note that incidence of stroke is much more related to mortality than to incidence of CeVD as a whole.

### Conclusion

The excess relative risk turned out to be quite different for the endpoint of CeVD incidence compared to stroke incidence and CeVD mortality, see Table 2. In contrast to certain cancer types, we believe this to be quite natural for cerebrovascular diseases. The first diagnosis of CeVD incidence may encompass a variety of atherosclerotic plaques, differing for example in size, site and composition. As the morphology affects further progression and clinical presentation [53], first acute complications are not necessarily related to the first incidence of CeVD.

Individual results for each endpoint compare well with findings from previous studies of the Mayak Worker Cohort and with results from other cohorts. For CeVD incidence, a dose-response relationship sub-linear for low doses was favored, see Fig 2, and younger age was significantly related to higher radiation risk. Similarly, in the analysis on stroke incidence, risk was significantly enhanced for workers below about 60 years. We believe the stroke analysis to be especially meaningful as stroke diagnosis is very accurate. For CeVD mortality, risk was not significantly enhanced.

## Supporting Information

S1 Appendix Details on the baseline and the models for the dose-response relationship. (PDF)

S1 Table Deviances for different ERR models of the dose response for CeVD incidence in reactor workers. (PDF)

S2 Table Deviances for different ERR models of the dose response for stroke incidence, each modified by a decreasing step in age attained. (PDF)

S3 Table Best parameter estimates and 95% confidence intervals for the ERR models of the dose-response relationship that passed the likelihood-ratio test. (PDF)

S4 Table Parameters for modification of the external dose response by gender and various risk factors for CeVD incidence in reactor workers, based on an LNT model and no lag time. (PDF)
