# Supplementary material for: Cerebrovascular Diseases in Workers at Mayak PA: The Difference in Radiation Risk between Incidence and Mortality
Source: PLoS One. 2015 May 1;10(5):e0125904. doi: 10.1371/journal.pone.0125904 (PMC4416824; doi:10.1371/journal.pone.0125904)
Supplement: S1 Appendix — (PDF) [file pone.0125904.s001.pdf]

A Appendix

A.1 Notation

To keep the tables in the supplementary material compact, estimates with a maximum likelihood estimation θ and a likelihood interval of $[\theta - \sigma_2, \theta + \sigma_1]$ are written as $\theta^{+\sigma_1}_{-\sigma_2}$.

A.2 The Baseline

A.2.1 The baseline template

We parameterized the baseline hazard h_0 for each gender separately by:

$$\begin{aligned} h_0 &= 10^{-5} e^{\psi_{\text{cat}} + \psi_{\text{time}} + \psi_{\text{emigration}}} \quad \text{(A1)} \\ \psi_{\text{cat}} &= \psi_{\text{smoking}} + \psi_{\text{drinking}} + \psi_{\text{bmi}} + \psi_{\text{blood pressure}} + \psi_{\text{graduation}} + \psi_{\text{plant}} \\ \psi_{\text{time}} &= \psi_{\text{age}} + \psi_{\text{birth}} + \psi_{\text{calendar}} + \psi_{\text{employment}} \end{aligned}$$

Summands in ψ_{cat} evaluate to zero for non-smoker, non-drinker, for persons with normal body mass index, normal blood pressure, secondary special education and workplace at the reactors. For other persons, the corresponding summand was determined by the fit. The other functions were defined as

$$\begin{aligned} \psi_{\text{age}} &= \psi_0 + \psi_1 \log \frac{a}{60} + \psi_2 \log^2 \frac{a}{60} + \sum_i \alpha_i \log^2 \frac{a}{\vartheta_{\alpha,i}} \cdot \Theta(a - \vartheta_{\alpha,i}) \\ \psi_{\text{birth}} &= \beta_1 \frac{b - 1900}{10} + \beta_2 \frac{(b - 1900)^2}{100} \quad \text{(A2)} \\ \psi_{\text{calendar}} &= \sum_i \gamma_i \frac{\text{LT}(b + a - \vartheta_{\gamma,i})}{10} \\ \psi_{\text{employment}} &= \delta_1 \frac{f - 1950}{10} + \delta_2 \frac{(f - 1950)^2}{100} \\ \psi_{\text{emigration}} &= \epsilon \cdot \Theta(b + a - m) \end{aligned}$$

Here, a, b, f and m denote age, birth date, date of first employment, and date of emigration from Ozyorsk, respectively. Lowercase Greek symbols are parameters determined by the fitting procedure. We have applied the Heaviside step function $\Theta(t)$ and a function $\text{LT}(t)$:

$$\Theta(t) = \begin{cases} 0 & \text{for } t < 0 \\ 1 & \text{for } t \geq 0 \end{cases} \qquad \text{LT}(t) = \begin{cases} 0 & \text{for } t < 0 \\ t & \text{for } t \geq 0 \end{cases} \quad \text{(A3)}$$

Depending on the endpoint and gender under consideration, only a subset of the parameters introduced in this template significantly deviated from zero. These were found by iterative testing and maintained for the analysis. In doing so, the dose response was parameterized by an excess relative risk model:

$$h = h_0(1 + ERR_{\text{ext}})(1 + ERR_{\text{int}}) \quad \text{(A4)}$$

where h denotes the hazard function and an LNT dose-response relationship was applied to ERR_{ext} with a lag-time of 10 years. In contrast, ERR_{int} may take some discrete value depending on the surrogate class of internal exposure and was retained only if significantly deviating from zero.

For convenience, we define here also the excess absolute risk (EAR) model:

$$h = h_0 + EAR_{\text{ext}} + EAR_{\text{int}} \quad \text{(A5)}$$

sex	male (75%), female (25%)
smoking	smoker (55%), non-smoker (38%), unknown information (7%)
drinking	drinker (69%), non-drinker (19%), unknown information (12%)
body mass index	$<18.5\,kg/m^2$ (1%), normal (58%), $\geq 25\,kg/m^2$ (14%), unknown information (27%)
blood pressure	normal (75%), $>140/90$ mmHg (14%), unknown information (11%)
graduation	primary education (9%), secondary education (16%), secondary special education (38%), entered higher education (16%), unknown information (21%)
plant	reactors (23%), radiochemical plant (42%), plutonium plant (35%)

Table A1. Frequency table of the categorical variables. The cohort has been defined as in the CeVD mortality analyis.

A.2.2 Non-radiation risk factors

The frequency of categorical baseline parameters can be found in table A1. The corresponding best fit results and 95% confidence intervals are presented in table A2. According to eq. (A1) the parameters have to be exponentiated to obtain relative risks. Thus, for stroke incidence, the risk for male smokers is observed to be $e^{0.21} = 1.23$ times the risk of male non-smokers. We employed in the analyses only parameters significantly deviating from zero. An exception are the parameters related to the categories of missing information. Those were kept as free parameters if the corresponding attribute contained a significant category. Best estimates and confidence intervals were derived by fixing all non-significant parameters to zero except the variable under consideration.

For CeVD incidence, the risk factors seem to differ in their effect compared to stroke incidence and CeVD mortality. For example, primary education was associated with a 1.36 fold increase in risk in males for CeVD mortality but with a 0.74 fold decrease in risk for CeVD incidence. The decrease, however, was related only to male reactor workers.

For stroke incidence and CeVD mortality, the risk factors of smoking, overweight and hypertension could be confirmed in many analyses. Higher education seemed to be negatively associated with risk at least for males. Work plant was not a significant covariable.

Other baseline parameters are shown in table A3. The dependence on age, calendar year and birth year is correlated and the corresponding variables may not be interpreted separately. Still, the position of the kinks in the calendar year dependence, $\vartheta_{\gamma,1}$, $\vartheta_{\gamma,2}$, can be roughly recovered in the crude incidence rates, see the left panel of fig. 1 in the main text. Surprisingly, for mortality in female workers, risk was found to be lower if hired in the early years of operation. While migration was not significant in males, the apparent risk of mortality in emigrated females was about 0.6 times the risk of Ozyorsk inhabitants. For a discussion of possible reasons see the methods section of the main text.

Attribute	Category	CeVD incidence		Stroke incidence		CeVD mortality	
		M	F	M	F	M	F
ψ_{smoking}	smoker	$0.07^{+0.15}_{-0.15}$	$-0.11^{+0.45}_{-0.52}$	$\mathbf{0.21}^{+0.16}_{-0.15}$	$0.23^{+0.38}_{-0.43}$	$\mathbf{0.22}^{+0.16}_{-0.16}$	$\mathbf{0.42}^{+0.38}_{-0.42}$
ψ_{drinking}	drinker	$-0.01^{+0.33}_{-0.30}$	$0.07^{+0.21}_{-0.21}$	$-0.15^{+0.41}_{-0.37}$	$-0.16^{+0.19}_{-0.20}$	$-0.02^{+0.32}_{-0.29}$	$-0.09^{+0.20}_{-0.20}$
ψ_{bmi}	$<18.5\,\text{kg/m}^2$	$0.01^{+0.55}_{-0.67}$	$\mathbf{1.51}^{+0.98}_{-1.43}$	$-0.43^{+0.68}_{-0.87}$	$-0.26^{+0.98}_{-1.43}$	$0.10^{+0.68}_{-0.87}$	$-0.35^{+0.98}_{-1.43}$
	$\geq 25\,\text{kg/m}^2$	$-0.01^{+0.16}_{-0.17}$	$-0.04^{+0.24}_{-0.25}$	$0.04^{+0.17}_{-0.17}$	$\mathbf{0.23}^{+0.20}_{-0.20}$	$0.12^{+0.19}_{-0.19}$	$0.21^{+0.22}_{-0.22}$
$\psi_{\text{blood press.}}$	$>140/90\,\text{mmHg}$	$0.05^{+0.16}_{-0.16}$	$-0.13^{+0.33}_{-0.36}$	$\mathbf{0.31}^{+0.15}_{-0.16}$	$\mathbf{0.41}^{+0.24}_{-0.26}$	$\mathbf{0.28}^{+0.16}_{-0.16}$	$0.21^{+0.24}_{-0.26}$
$\psi_{\text{graduation}}$	primary	$\mathbf{-0.30}^{+0.19}_{-0.20}$	$-0.03^{+0.25}_{-0.27}$	$0.08^{+0.19}_{-0.20}$	$0.13^{+0.22}_{-0.22}$	$\mathbf{0.31}^{+0.22}_{-0.23}$	$\mathbf{0.40}^{+0.22}_{-0.23}$
	secondary	$-0.03^{+0.15}_{-0.16}$	$-0.03^{+0.23}_{-0.23}$	$0.08^{+0.16}_{-0.17}$	$-0.05^{+0.21}_{-0.22}$	$\mathbf{0.28}^{+0.20}_{-0.20}$	$0.05^{+0.27}_{-0.28}$
	higher	$-0.06^{+0.15}_{-0.15}$	$-0.19^{+0.32}_{-0.35}$	$\mathbf{-0.39}^{+0.16}_{-0.16}$	$0.09^{+0.29}_{-0.31}$	$\mathbf{-0.36}^{+0.20}_{-0.20}$	$0.23^{+0.29}_{-0.31}$
ψ_{plant}	radiochemical			$0.09^{+0.13}_{-0.13}$	$0.04^{+0.18}_{-0.18}$	$-0.02^{+0.13}_{-0.14}$	$-0.05^{+0.18}_{-0.18}$
	plutonium			$-0.01^{+0.14}_{-0.14}$	$-0.04^{+0.19}_{-0.19}$	$0.11^{+0.14}_{-0.15}$	$0.04^{+0.18}_{-0.18}$

Table A2. Best estimates of the baseline parameters associated with categorical data and their 95% confidence intervals. The parameters for non-smoker, non-drinker, normal body mass index, normal blood pressure, for secondary special education and reactor workers are defined to be zero and are not shown. The parameters corresponding to unknown information are not shown either. Significant parameters are marked bold. In the related analyses, external doses were accounted for by applying an LNT model with a lag-time of 10 years.

Attribute	Var.	CeVD incidence		Stroke incidence		CeVD mortality	
		M	F	M	F	M	F
ψ_{age}	ψ_0	10.5	10.8	5.8	5.9	4.9	4.5
	ψ_1	-14.8	-8.9	6.9	6.6	9.0	11.0
	ψ_2	-21.0	-19.7			2.6	
ψ_{birth}	β_1	-2.4	-2.2	0.27		-0.00	
	β_2	-0.15				0.05	
ψ_{calendar}	γ_1	2.5	2.6	-0.38		-0.49	
	γ_2	1.1					
	$\vartheta_{\gamma,1}$	1965.6	1970.3	1994.1		1997.7	
	$\vartheta_{\gamma,2}$	1979.7					
$\psi_{\text{employment}}$	δ_1						0.25
$\psi_{\text{emigration}}$	ϵ						-0.50

Table A3. Best estimates of the baseline parameters not associated to categorical data. Only parameters significantly different from zero, are entered into the table. In the related analyses, external doses were accounted for by applying an LNT model with a lag-time of 10 years.

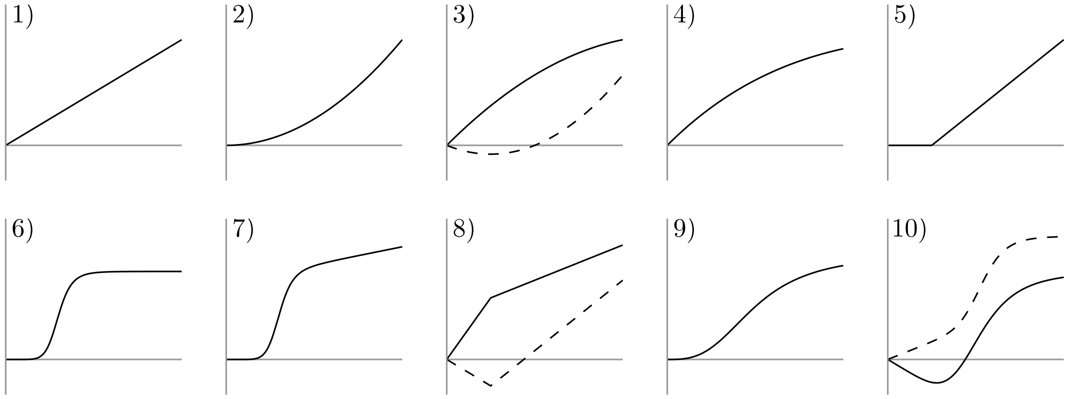

Figure A1. Typical shapes of the functions tested as dose response. Additional dashed lines show alternative shapes for some of the functions. The functions are called 1) LNT 2) Quadratic 3) Linear-quadratic 4) Linear-exponential 5) Linear-threshold 6) Step 7) Step-linear 8) Two-line-spline 9) Sigmoid 10) Hormesis

A.3 Functions of the dose response

We analyzed the dose-response relationship with the following ten functions, which are sketched in fig. A1.

$$\begin{aligned}
&\text{1) LNT:} && ERR_{\text{ext}}(d) = \lambda\, d && \text{(A6)}\\
&\text{2) Quadratic:} && ERR_{\text{ext}}(d) = \lambda\, d^2 \\
&\text{3) Linear-quadratic:} && ERR_{\text{ext}}(d) = \lambda_1\, d + \lambda_2\, d^2 \\
&\text{4) Linear-exponential:} && ERR_{\text{ext}}(d) = \lambda_1\, d \cdot \exp(-\lambda_2\, d) \\
&\text{5) Linear-threshold:} && ERR_{\text{ext}}(d) = \lambda\ \mathrm{LT}(d-\vartheta) \\
&\text{6) Step:} && ERR_{\text{ext}}(d) = \lambda_0 - \frac{\lambda_0}{1+\left(\frac{d}{\vartheta}\right)^8} \\
&\text{7) Step-linear:} && ERR_{\text{ext}}(d) = \lambda_0 - \frac{\lambda_0}{1+\left(\frac{d}{\vartheta}\right)^8} + \lambda_2\ \mathrm{LT}(d-\vartheta) \\
&\text{8) Two-line-spline:} && ERR_{\text{ext}}(d) = \lambda_0\, d + \lambda_1\ \mathrm{LT}(d-\vartheta) \\
&\text{9) Sigmoid:} && ERR_{\text{ext}}(d) = \lambda_0 - \frac{\lambda_0}{1+\left(\frac{d}{\vartheta}\right)^{\lambda_1}} \\
&\text{10) Hormesis:} && ERR_{\text{ext}}(d) = \lambda_0 - \frac{\lambda_0 + \lambda_2\, d}{1+\left(\frac{d}{\vartheta}\right)^{\lambda_1}}
\end{aligned}$$

Here, we used the function LT, see eq. (A3). The hormesis function was introduced in ref. [1]. For the same reasons outlined already in ref. [2], a smooth step function was used instead of an instantaneous one. We adopted the slope of the step function, $\lambda_1 = 8$, which may approximately resemble the smoothing effect of dose uncertainty.

References

1. Brain P, Cousens R (1989) An equation to describe dose responses where there is stimulation of growth at low doses. Weed Research 29: 93–96.

2. Simonetto C, Azizova TV, Grigoryeva ES, Kaiser JC, Schöllnberger H, et al. (2014) Ischemic heart disease in workers at Mayak PA: Latency of incidence risk after radiation exposure. PLoS ONE 9: e96309.
