# Supplementary material for: Cerebrovascular Diseases in Workers at Mayak PA: The Difference in Radiation Risk between Incidence and Mortality
Source: PLoS One. 2015 May 1;10(5):e0125904. doi: 10.1371/journal.pone.0125904 (PMC4416824; doi:10.1371/journal.pone.0125904)
Supplement: S1 Table — (PDF) [file pone.0125904.s002.pdf]

	LNT	Quadra-tic	Linear-quad.	Linear-exp.	Linear-thresh.	Step	Step-linear	2-line-spline	Sigmoid	Horm.
no	-29.8	-33.7	-34.0	-34.0	-33.5	-23.7	-33.8	-33.9	-33.9	-34.0
exp.	**-34.6**	-36.2	-37.3	-37.2	-37.9	-28.5	-38.0	-37.9	-37.4	-37.4
step	**-35.8**	**-37.5**	-38.3	-38.4	-38.4	-28.9	-39.0	-38.5	**-38.4**	-38.4

Table S1. Deviances for different ERR models of the dose response for CeVD incidence in reactor workers. In addition to the unmodified analysis, results from effect modification exponential and with a step in age attained are presented. The deviance is presented as the difference from the baseline's deviance. No time lagging was applied. Models that passed the likelihood-ratio test are marked bold.
