# Supplementary material for: Cerebrovascular Diseases in Workers at Mayak PA: The Difference in Radiation Risk between Incidence and Mortality
Source: PLoS One. 2015 May 1;10(5):e0125904. doi: 10.1371/journal.pone.0125904 (PMC4416824; doi:10.1371/journal.pone.0125904)
Supplement: S2 Table — (PDF) [file pone.0125904.s003.pdf]

LNT	Quadratic	Linear-quadratic	Linear-exponential	Linear-threshold	Step
**-7.4**	**-8.9**	-10.1	-7.5	-10.9	**-12.3**

Table S2. Deviances for different ERR models of the dose response for stroke incidence, each modified by a decreasing step in age attained. The deviance is presented as the difference from the baseline's deviance. No time lagging was applied. Models that passed the likelihood-ratio test are marked bold.
