# Supplementary material for: Cerebrovascular Diseases in Workers at Mayak PA: The Difference in Radiation Risk between Incidence and Mortality
Source: PLoS One. 2015 May 1;10(5):e0125904. doi: 10.1371/journal.pone.0125904 (PMC4416824; doi:10.1371/journal.pone.0125904)
Supplement: S3 Table — (PDF) [file pone.0125904.s004.pdf]

Endpoint	dose resp.	age mod.	parameter values
CeVD inc.	LNT	exponential	λ = 0.25 (0.10; 0.43) Gy^{-1}, μ' = -3.7 (-7.7; -0.3)
	LNT	step down	λ = 0.39 (0.24; 0.59) Gy^{-1}, μ = 64.4 (63.2; 70.1) years
	Quadratic	step down	λ = 0.16 (0.10; 0.24) Gy^{-2}, μ = 68.5 (63.3; >83) years
	Sigmoid	step down	λ_0 = 575 (1.0; ∞), λ_1 = 1.6 (1.0; 2.5), ϑ = 120 (1.5; ∞) Gy, μ = 68.4 (63.2; 81.1) years
Stroke inc.	LNT	step down	λ = 0.34 (0.05; 0.76) Gy^{-1}, μ = 48.8 (41.1; 54.6) years
			**or** λ = 0.14 (0.03; 0.28) Gy^{-1}, μ = 64.2 (57.8; 69.5) years
	Quadratic	step down	λ = 0.18 (0.04; 0.38) Gy^{-2}, μ = 49.1 (45.4; 53.9) years
			**or** λ = 0.06 (0.01; 0.15) Gy^{-1}, μ = 62.9 (<55; 69.0) years
	Step	step down	λ_0 = 1.8 (0.5; 4.6), ϑ = 1.9 (1.1; 2.8) Gy, μ = 49.3 (46.1; 54.5) years
			**or** λ_0 = 1.0 (0.3; 2.4), ϑ = 2.1 (1.4; 2.9) Gy, μ = 58.7 (56.8; 65.9) years

Table S3. Best parameter estimates and 95% confidence intervals for the ERR models of the dose-response relationship that passed the likelihood-ratio test.
