# Supplementary material for: Cerebrovascular Diseases in Workers at Mayak PA: The Difference in Radiation Risk between Incidence and Mortality
Source: PLoS One. 2015 May 1;10(5):e0125904. doi: 10.1371/journal.pone.0125904 (PMC4416824; doi:10.1371/journal.pone.0125904)
Supplement: S4 Table — (PDF) [file pone.0125904.s005.pdf]

Attribute	$\psi^{\mathrm{mod}}_{\mathrm{gender}}$	$\psi^{\mathrm{mod}}_{\mathrm{smoking}}$	$\psi^{\mathrm{mod}}_{\mathrm{drinking}}$	$\psi^{\mathrm{mod}}_{\mathrm{bmi}}$		$\psi^{\mathrm{mod}}_{\mathrm{blood\ pressure}}$
Category	female	smoker	drinker	$<18.5\,\mathrm{kg/m^2}$	$\geq 25\,\mathrm{kg/m^2}$	$>140/90\,\mathrm{mmHg}$
Value	$-1.1^{+1.6}_{-\infty}$	$-0.3^{+1.2}_{-1.0}$	$-0.3^{+5.1}_{-1.1}$	$-0.1^{+4.1}_{-\infty}$	$-1.6^{+1.9}_{-\infty}$	$-0.4^{+1.1}_{-3.1}$

Table S4. Parameters for modification of the external dose response by gender and various risk factors for CeVD incidence in reactor workers, based on an LNT model and no lag-time. Best estimates are presented together with their 95% confidence intervals. Ratios of the excess relative risk for persons with a specific risk factor to persons without may be calculated as $\exp(\psi^{\mathrm{mod}}_{\mathrm{cat}})$.
